# Management Model for the Evaluation of needs and Acquisition of Biomedical Equipment in Healthcare Institutions: Based on WHO Recommendations, and National Regulations

**DOI:** 10.1007/s10916-025-02329-2

**Published:** 2026-04-05

**Authors:** Yeimy Liseth Quintana Villamizar, Lina Mayerly Cruz Parra, Fabian Alonso Carvajal Arevalo, Yeison Andrés López Lozano

**Affiliations:** 1 Socio-Economics and Regional Development (GIESD), Instituto Superior de Educación Rural (ISER), Pamplona, Norte de Santander Colombia; 2 Biomedical Research and Innovation Group (Gi2B), Faculty of Exact and Applied Sciences, Instituto Tecnológico Metropolitano, Medellín, Antioquia Colombia

## Abstract

This study aims to develop and validate a model for the evaluation of needs and acquisition of biomedical equipment in Colombian Health Care Institutions (IPS). Grounded in World Health Organization (WHO) recommendations and national regulations, the model integrates demographic, epidemiological, and institutional data to support informed decision-making and optimize resource allocation based on local healthcare needs. The model incorporates demographic, epidemiological, human resources, infrastructure, and equipment variables. It follows four phases: data collection, analysis, evaluation of alternatives, and implementation planning. Validation was conducted in two IPS with different complexity levels in Norte de Santander, using a web-based tool developed in Laravel 10. Both qualitative and quantitative usability assessments were carried out. The model was deployed through a web platform built with HTML, CSS, and Laravel 10, allowing automated identification of biomedical equipment needs and service gaps. It identified deficiencies by comparing institutional capacity against regulatory standards and applied a prioritization mechanism using the PDCA (Plan, Do, Check, Act) cycle. This supported structured acquisition plans aligned with institutional capabilities and accreditation requirements. Usability testing revealed high operational efficiency, intuitive navigation, and strong integration with healthcare planning processes. Evaluation time was reduced by 70%, and average accreditation compliance scores exceeded 4.4 out of 5. Validation was conducted in two healthcare institutions in a single Colombian region, which defines the scope and generalizability of the findings. Quantitative validation showed statistically significant improvements (*p* < 0.05) in compliance indicators and evaluation time reduction. The model demonstrated effectiveness in biomedical equipment planning within IPS, supporting evidence-based decisions and strategic resource use. Its modular and scalable design enables adaptation across diverse healthcare contexts, fostering equitable and efficient management of biomedical technology. The results provide consistent evidence of the model’s applicability and potential for broader implementation across healthcare institutions.

## Introduction

The article introduces a needs assessment model for acquiring biomedical equipment in Colombian healthcare institutions. The model is based on the WHO’s technical document and aligns with national regulations [1]. It aims to identify deficiencies in biomedical equipment across IPS (Healthcare Provider Institutions), considering demographic and epidemiological data, the availability of health services, and current equipment inventory. The goal is to optimize resource allocation through better planning and prioritization.

The model recommends comprehensive data collection, focusing on service requirements and equipment needs. Demographic and epidemiological data, as well as information on IPS services, define the target population, disease burden, and transfer risks.

Subsequently, the services provided by the Healthcare Institutions (IPS) are identified. For each service, the status of biomedical equipment is assessed. This process results in a list of the biomedical equipment needed to meet the demands of the identified services. Finally, the biomedical technology management of the IPS is analyzed on acquisition.

A web-based system was developed to create the modules required by the model for data collection and analysis. From wireframes generated in Figma, a functional system was built using HTML, CSS, and JavaScript, modularized and integrated into Laravel 10. Once populated with data on demographics, epidemiology, service availability, and biomedical equipment, the system identifies the equipment needed to meet IPS requirements. The results are organized into a template that consolidates essential information for project formulation.

The model was validated in real settings: first using Excel/VBA in a medium-complexity IPS, then through a web prototype in a low-complexity IPS in Norte de Santander, Colombia.

Health systems, particularly in low- and middle-income countries, often face significant challenges in identifying and prioritizing their biomedical equipment needs. These challenges are typically the result of fragmented data, limited technical resources, and a lack of standardized methodologies. In response to these issues, the World Health Organization (WHO) published the Medical Device Needs Assessment guidelines, which offer a structured approach for evaluating and planning medical device acquisition.

This article presents a comprehensive model designed to assess biomedical equipment needs in Colombian Health Care Provider Institutions (IPS), aligning with WHO recommendations and national health regulations. The model integrates demographic, epidemiological, and operational data to identify service gaps and prioritize equipment acquisition based on local health needs and institutional capacity.

To support its application, a web-based information system was developed using Laravel 10, incorporating wireframes designed in Figma and front-end technologies such as HTML, CSS, and JavaScript. The platform enables automated data analysis and generates structured reports to support project formulation for equipment acquisition.

The model was validated in two real-world healthcare settings in Norte de Santander, Colombia: a medium-complexity IPS using Excel and VBA, and a low-complexity IPS through a Laravel-based prototype. This article details the validation process, emphasizing improvements in usability, decision-making, and alignment with institutional planning processes. The aim of this study is to develop and validate a model for evaluating biomedical equipment needs and acquisition processes in Colombian healthcare institutions, integrating WHO recommendations and national regulatory criteria.

## Methodology

The model compares the IPS’s current resources with regulatory requirements, based on the area of influence. A web platform integrates various modules to collect and analyze data automatically. The methodology was structured in the following steps:

1. Comparative Analysis of Needs Assessment Methodologies for Biomedical Equipment Acquisition

A literature review was conducted, including the “Review of Management Models for Needs Assessment to Acquire Biomedical Equipment” study [1, 2]. Covered studies indexed in PubMed, Scopus, and WHO IRIS (2010–2024) related to medical device needs assessment and health technology management. Selection focused on methodological reports and WHO/agency documents, excluding opinion papers and non-health devices. Gaps were identified in integrating technology management with acquisition planning. A comparative analysis with global standards highlighted differences in criteria, metrics, and applicability (see Table 1).

**Table 1:** Comparison of needs assessment methodologies for biomedical equipment acquisition

Model	Main Approach	Evaluation Criteria
Review of Management Models for Needs Assessment to Acquire Biomedical Equipment	Review of existing models and identification of methodological gaps	Health technology management, needs assessment, impact on acquisition (7)
WHO Needs Assessment for Medical Devices	Needs assessment based on global standards	Availability, accessibility, safety, and cost (7)
Medical Equipment Integrity Assessment Model	Functionality analysis and failure risk assessment of medical devices	Equipment condition, vulnerability, cybersecurity (6)
HTA-Based Methodology for Medical Equipment Replacement	Cost-effectiveness evaluation in replacement decisions	Investment costs, impact on clinical processes (4)
Multi-Criteria Decision-Making (MCDM) Model	Decision-making and data analysis-based approach	Technical, economic, and operational factors(5)
Proposed Model	Integration of global standards with Colombian regulations	Local regulations, needs prioritization, financial feasibility

Subsequently, a comparative analysis was carried out with other methodological approaches presented in additional studies.[3–6], identifying significant differences in prioritization criteria, decision-making metrics, and applicability in different contexts as shown in Table 1. Since the acquisition of biomedical equipment is a global challenge, international standards were adjusted to the specific needs of the Colombian health system, ensuring alignment with local regulations and each institutional capability.

2. Standardization of Tools for Collecting Information on Items I–V

Seven modules (A–G) were defined:

- Module I (ID: A): Collection of demographic and epidemiological characteristics, providing the baseline information on IPS´s health service needs.
- Modules II and III (ID: B and C): Collection of availability of health services and medical equipment by services, this tool identifies baseline information on the availability of health services (ID: B), and baseline information on medical equipment (ID: C).
- Modules IV to VII (ID: D–E–F–G): Collection of data related to infrastructure, biomedical technology management, human resources, and economic information aspects.

**Fig. 1:**
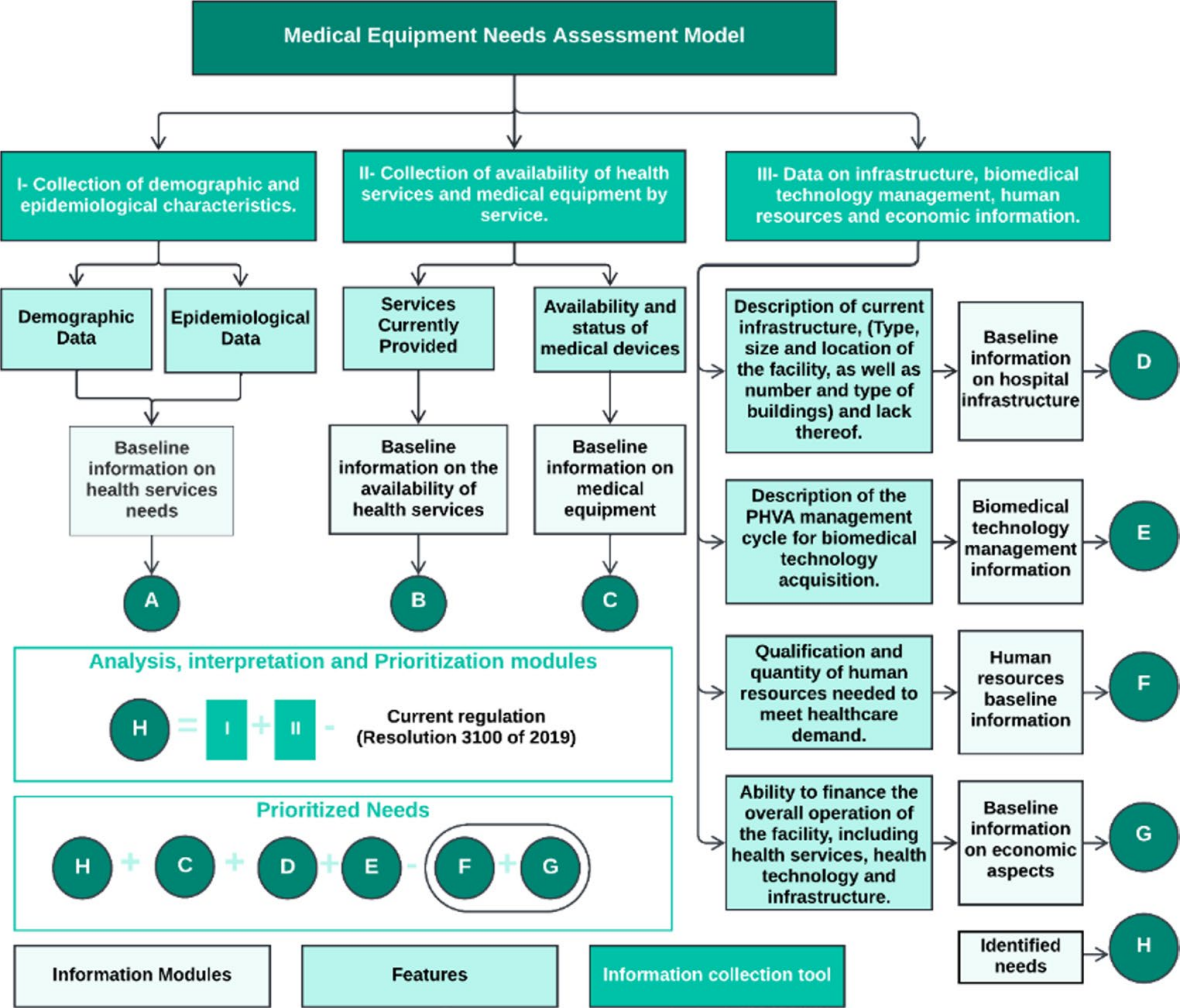
- Summary of Information Collection, Analysis, Interpretation, and Prioritization Modules. Source: Own elaboration.

These modules standardize the information for subsequent analysis, interpretation, and prioritization, as summarized in Fig. 1.

3. Data Analysis and Interpretation

Once data is collected, results from Modules I–III are compared with the requirements in Resolution 3100 of 2019. This generates the identifier ID: H, which reflects the gap between existing and required resources. Economic and human resource constraints and the institution’s technology management capacity (from Modules IV–VII) are then used to refine prioritization. This approach supports efficient allocation and a coherent equipment acquisition plan.

### Data Collection

According to the WHO Medical Device Needs Assessment document, the first part of the model consists of collecting demographic and epidemiological information on the area of influence of the IPS.

#### Demographic Data

Details regarding the size and characteristics of the population in the IPS’s area of influence are collected. It is recorded whether the population exceeds 100,000 inhabitants, and three prevalent risks are identified: earthquakes, floods, fires, landslides, erosion, pollution, immigration, violence, and displacement. Information on travel time, transportation modes, and proximity to referral centers is included. The percentage of males and females in the population, along with age distribution: 0–14 years and 15–59 years.

#### Epidemiological Data

**Fig. 2:**
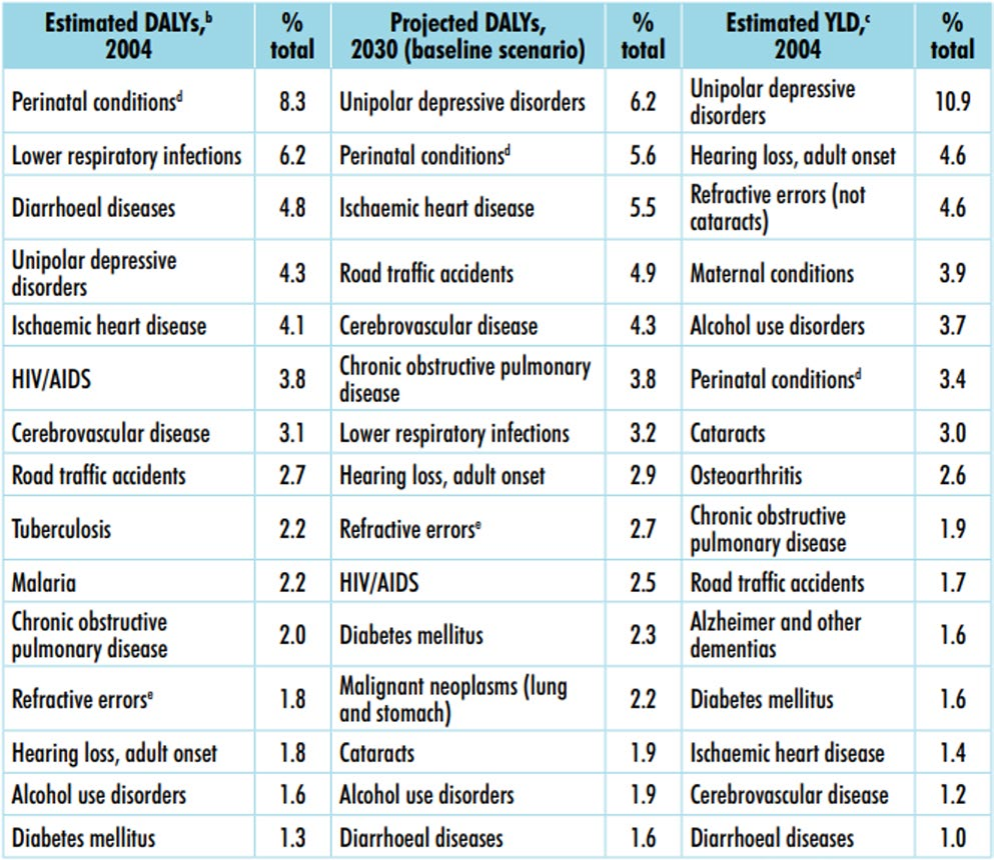
Estimated Disability-Adjusted Life Years (DALYs) and Years Lived with Disability (YLDs) for 15 of the leading causes of disease burden worldwide, in 2004 and 2030. Source: [7].

To determine the necessary biomedical equipment, the model leverages findings from the World Health Organization’s Priority Medical Devices (PMD) project. Epidemiological data is collected from the municipalities within the area of influence, revealing the primary disease burden and priority conditions, based on the 15 conditions that contribute most significantly to the global disease burden (as measured by DALYs in 2004 and projections for 2030). It ensures procurement aligns with health priorities (see Fig. 2).

#### Health Services Data

Next, information is collected on the health services available at the IPS. This process begins with the identification of the services currently offered and then the availability and condition of biomedical equipment are evaluated, following the guidelines of Resolution 3100 of 2019 [8].

Data is collected on existing services and associated equipment, evaluated using Resolution 3100. Each service undergoes a short assessment to identify gaps and renewal needs.

**Fig. 3:**
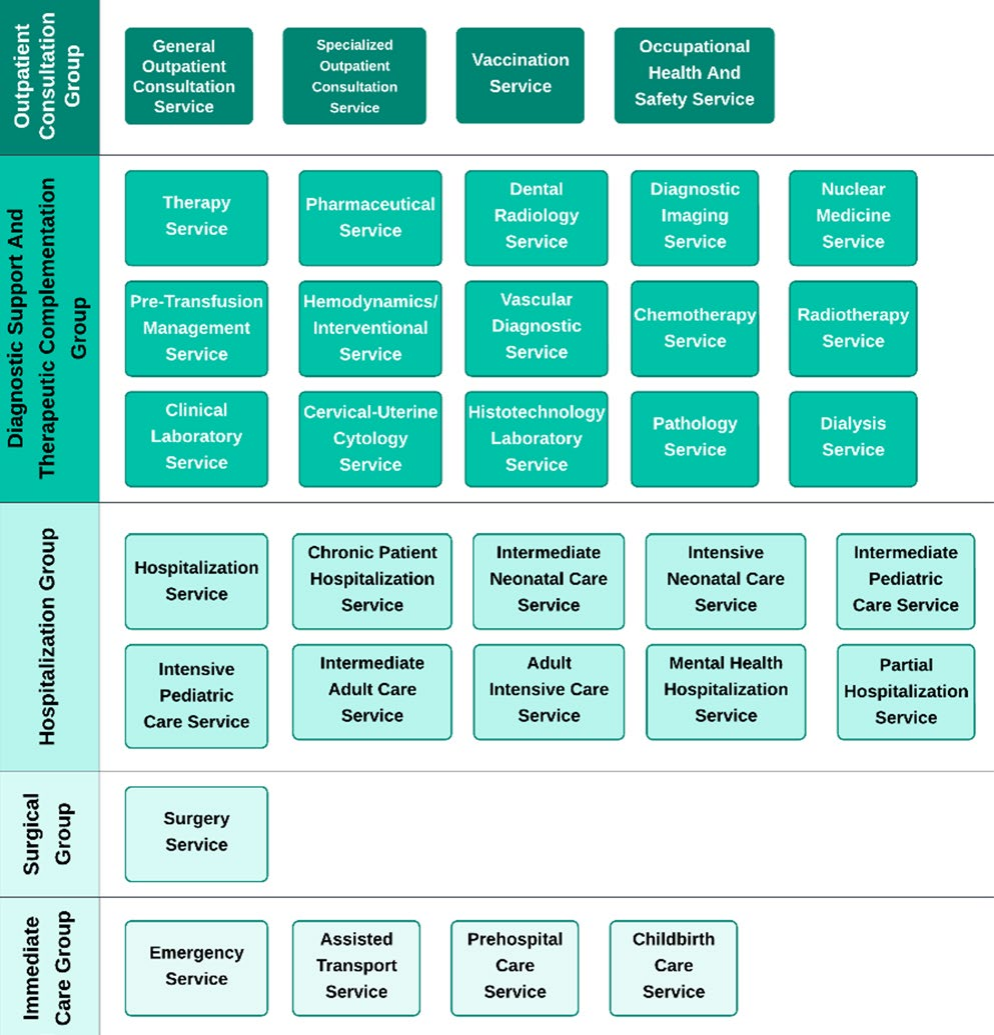
Availability of health services in the IPS, illustrating the assessment of the state of biomedical equipment associated with each service, by Resolution 3100 of 2019. Source: Own elaboration.

An example of the services to be evaluated according to the resolution is shown in Fig. 3.

### Development of Data Collection and Analysis Tool

The web platform was developed using a SCRUM-based approach with two-week sprints. Its architecture, implemented in Laravel 10 with a MySQL database, integrates HTML, CSS, and JavaScript modules for structured data collection and automated analysis. The development followed best practices for web-based medical management systems [9–11].

#### Data Analysis

Once the data is collected using the instruments described, the next step is to analyze and interpret it to define equipment needs. In this stage, the model characterizes service provision based on regulatory requirements, population size, age distribution, and regional risks. It also prioritizes causes of morbidity using WHO’s Priority Medical Devices Project as a reference. This analysis defines the biomedical equipment needed per service, improving alignment with clinical demand and enabling efficient planning based on institutional complexity and capacity.

The data analysis is performed as follows tools:

- Tool I: Identify the necessary biomedical services and equipment, considering the demographic and epidemiological characteristics, using the equipment availability matrix.
- Tool II: Compares the current equipment with that required according to Resolution 3100 of 2019, to identify the equipment that must be renewed in each service.
- Tool III: Establishes priorities for the acquisition of equipment, evaluating the infrastructure, financial resources, and technological management of the IPS.

4. Analysis of Alternatives

This module focuses on prioritizing needs by ranking the equipment to be acquired based on their clinical importance, impact on processes, and technical and economic feasibility. The methodology evaluates the availability of funding and resources for replacements or acquisitions.

The PDCA matrix ranks equipment by clinical importance, impact, and feasibility (see Table 2). Equipment prioritization was based on expert consensus through structured discussions, following principles similar to the Delphi approach to ensure consistency and reproducibility.

**Table 2:** PDCA matrix is used for prioritizing alternatives in the acquisition of biomedical equipment. Source: own elaboration

Financing or Resources Needed for the Change	Likely Impact of the Change	
	Low	High
Few	Easy target: wait	Advantageous: go ahead!
Many	Hold off or wait	Problematic: wait

The prioritization matrix was refined through structured expert discussions inspired by the Delphi method, ensuring reproducibility and methodological transparency. Experts participated in two iterative feedback rounds until reaching consensus on prioritization criteria.

5. Development of the Implementation Plan

Once the equipment needs have been identified and prioritized, an action plan is developed for their acquisition. According to WHO, this plan includes [12].

- Objectives and goals: Clearly defined and aligned with the identified needs.
- Timeline: Specific dates for each phase of the process.
- Responsible parties: Identification of key stakeholders involved in the implementation.
- Resources: A detailed list of the financial, human, and technological resources required.
- Monitoring mechanisms: Indicators and methods to track progress and evaluate the plan’s effectiveness.

The implementation plan serves as a tool to optimize resource allocation and improve the quality of health services.

## Results

### Web Platform Deployment and Tools Overview

**Fig. 4:**
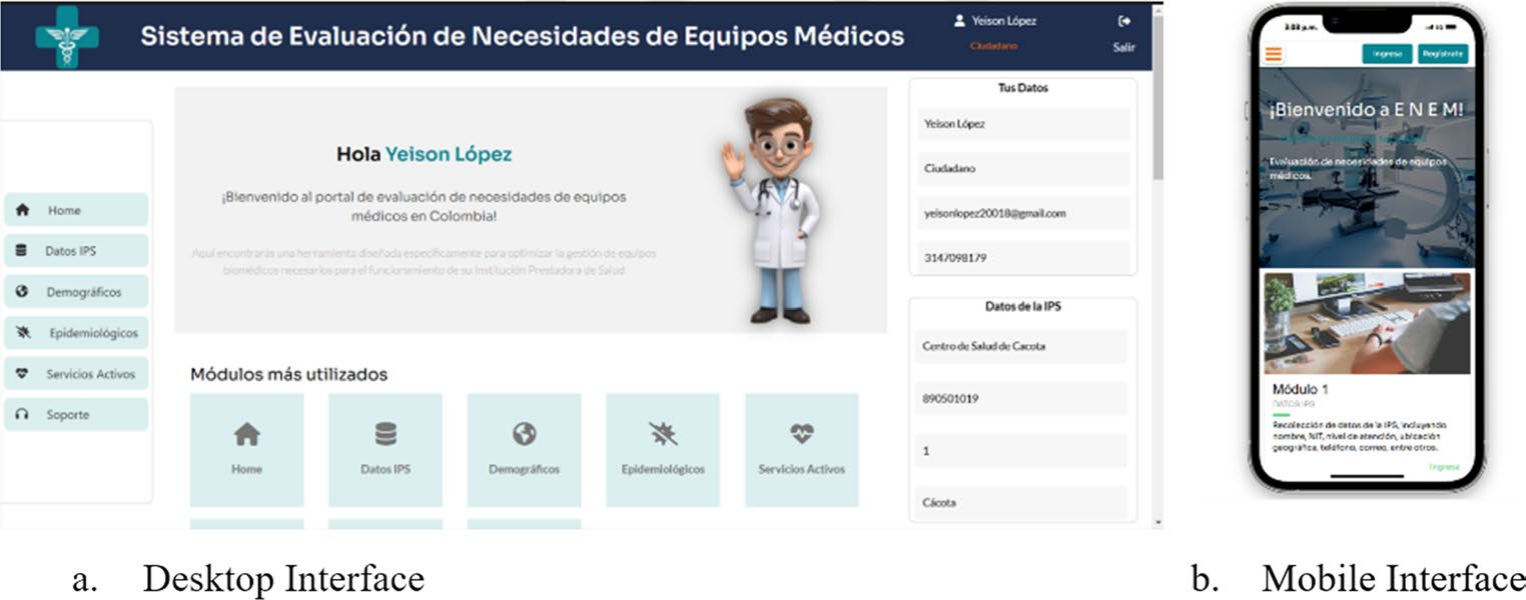
Home-Start screen of data collection and analysis software Source: Own elaboration

The Laravel-based platform consists of modules covering demographic profiling, equipment analysis, prioritization, and project formulation. The modular structure reflects each stage of the biomedical equipment needs assessment: demographic and epidemiological profiling (Tool I), health services and equipment availability analysis (Tool II), prioritization of biomedical equipment (Tool III), and project formulation. Figure 4 displays the initial interface for desktop and mobile devices.

1. Demographic and Epidemiological Profiling (Tool I)

Tool, I collect data to determine service demand. It is composed of four modules (Table 3), focusing on demographic characterization and epidemiological profiling.

**Table 3:** Data collection module. Source: own elaboration

Module	Description
I: Home	Access to the portal, user data review, and navigation across modules.
II: IPS Data Collection	Collection of institutional data such as name, NIT, care level, geographic location, phone number, and email.
III: Demographic Data	Characterization of the population served, including age, gender, population size, and assessment of regional risks (e.g., floods, landslides, earthquakes, fires).
IV: Epidemiological Data	Recording the primary causes of morbidity and the most prevalent diseases in the area of influence.

### Demographic Data

- Population: Institutions serving < 100,000 people are categorized as low to medium complexity (levels 1–2). Higher population suggests the need for extended service coverage.
- Risk Profile: Users select the three most relevant regional risks (e.g., floods, landslides, violence), which determine the services the IPS should offer. Table 4 presents service recommendations according to risk types.

**Table 4:** Health services recommended according to risks factors. Source: own elaboration

Services according to risks			
Risk	**Service**	**Risk**	**Service**
Floods	Intermediate and Intensive Care Service	**Contamination**	Specialized Outpatient Service
Earthquakes/Landslides	Diagnostic Imaging Service	**Displacement**	Therapy Service
Violence	Pre-transfusion Management Service	**Fires**	Intermediate and Intensive Care Service

### Epidemiological Data

Health services are determined based on studies emphasizing the importance of specific services for emergencies related to various risks: diagnostic imaging for trauma from earthquakes and landslides, specialized hospitalization for fires, and drowning care for floods [9–11, 13]. The model uses this data to identify essential IPS services according to the risks.

For epidemiological data, the DMP project identifies the 15 diseases contributing most to the global burden of morbidity. Users select the top 5 causes of morbidity in the area of influence based on the Region’s Health Situation Analysis.

The priority devices identified in the WHO document on essential medical devices [7] are used. The availability matrix and the study of priority conditions and medical devices help identify the required equipment for each disease, as shown in Table 5.

**Table 5:** Biomedical equipment according to the epidemiological profile of the IPS. Source: own elaboration

Biomedical Equipment Based on the Epidemiological Profile			
Morbidity Cause	**Required Biomedical Equipment**	**Morbidity Cause**	**Required Biomedical Equipment**
Tuberculosis	Laboratory Equipment	**Neurological Disease**	MRI Scanner/CT Scanner
	X-Ray Machine		Electroencephalogram
HIV/AIDS	Laboratory Equipment	**Diarrheal Disease**	N/A
Malaria	Laboratory Equipment	**Diabetes Mellitus**	Laboratory Equipment
Respiratory Infections	Pulse Oximeter	**Obstructive Pneumopathy**	Nebulizer
	Mechanical Ventilation		Spirometer
	Vital Signs Monitor		Mechanical Ventilation Equipment
Cataracts	Ophthalmology Equipment	**Depressive Disorders**	Neurological Stimulation Equipment
Perinatal Conditions	Crash Cart	**Malignant Neoplasms**	Radiotherapy Equipment
	Ultrasound scanner		Chemotherapy Equipment
	Mechanical Ventilation		PET equipment
	Incubator		CT Scanner
	Portable Incubator		MRI Scanner
	Vital Signs Monitor		Mammography Machine
Traffic Accident	Ultrasound scanner	**Ischemic Heart Disease**	Crash Cart
	Mechanical Ventilation Equipment		Blood Pressure Monitor
	Crash Cart		Pulse Oximeter
	Anesthesia Machines		Electrocardiogram
	Surgical Equipment		
	Ultrasound scanner

2. Assessment of Services and Equipment (Tool II)

**Fig. 5:**
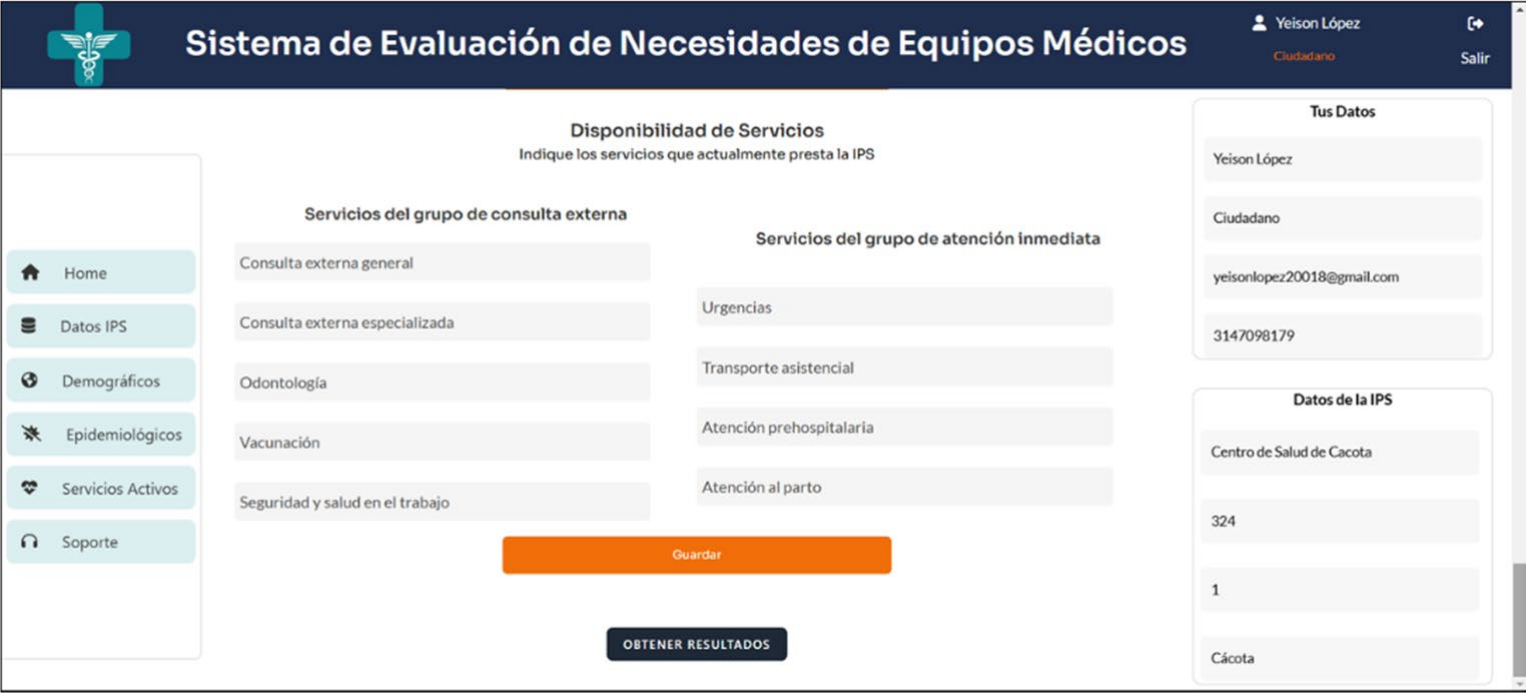
Interface for service health data collection. Source: Own elaboration

Tool II evaluates existing services and associated equipment using Modules V, VI, and VII (Fig. 5; Table 6), aligned with Resolution 3100 of 2019. The system automatically determines equipment gaps by comparing demographic demands and current service provision.

**Table 6:** Description of service and equipment data modules Source: own elaboration

Module	Description
V: Health Service Availability	Identification of health services provided by the IPS (e.g., emergency care, hospitalization, surgery, outpatient services) following Resolution 3100 of 2019 (9).
VI: Analysis of Medical Equipment Availability	Assessment of the availability and adequacy of medical equipment for each identified health service.
VII: Results Analysis	Recommendations for introducing new services and acquiring necessary biomedical equipment. Analysis of existing equipment and suggestions for upgrades or replacements.

The analysis identifies the missing services based on demographic needs, the equipment gaps related to prevalent diseases, the devices that require renewal, and the current status of infrastructure, human resources, and technology management.

3. Prioritization Matrix and Gap Analysis (Tool III)

The tool identifies the priority needs for biomedical equipment in IPS based on three key aspects:

1. Existence of a technological management system for the acquisition of equipment.
2. Availability of financial resources earmarked for the purchase of equipment.
3. Capacity for growth in hospital infrastructure, with the potential to offer new services.

The valuation considers equipment quantity, strategic impact, and feasibility. Users reflect on impact and change capacity to support decision-making.

4. Standardization of the Model Based on the WHO-Recommended Medical Device Needs Evaluation Criteria

**Fig. 6:**
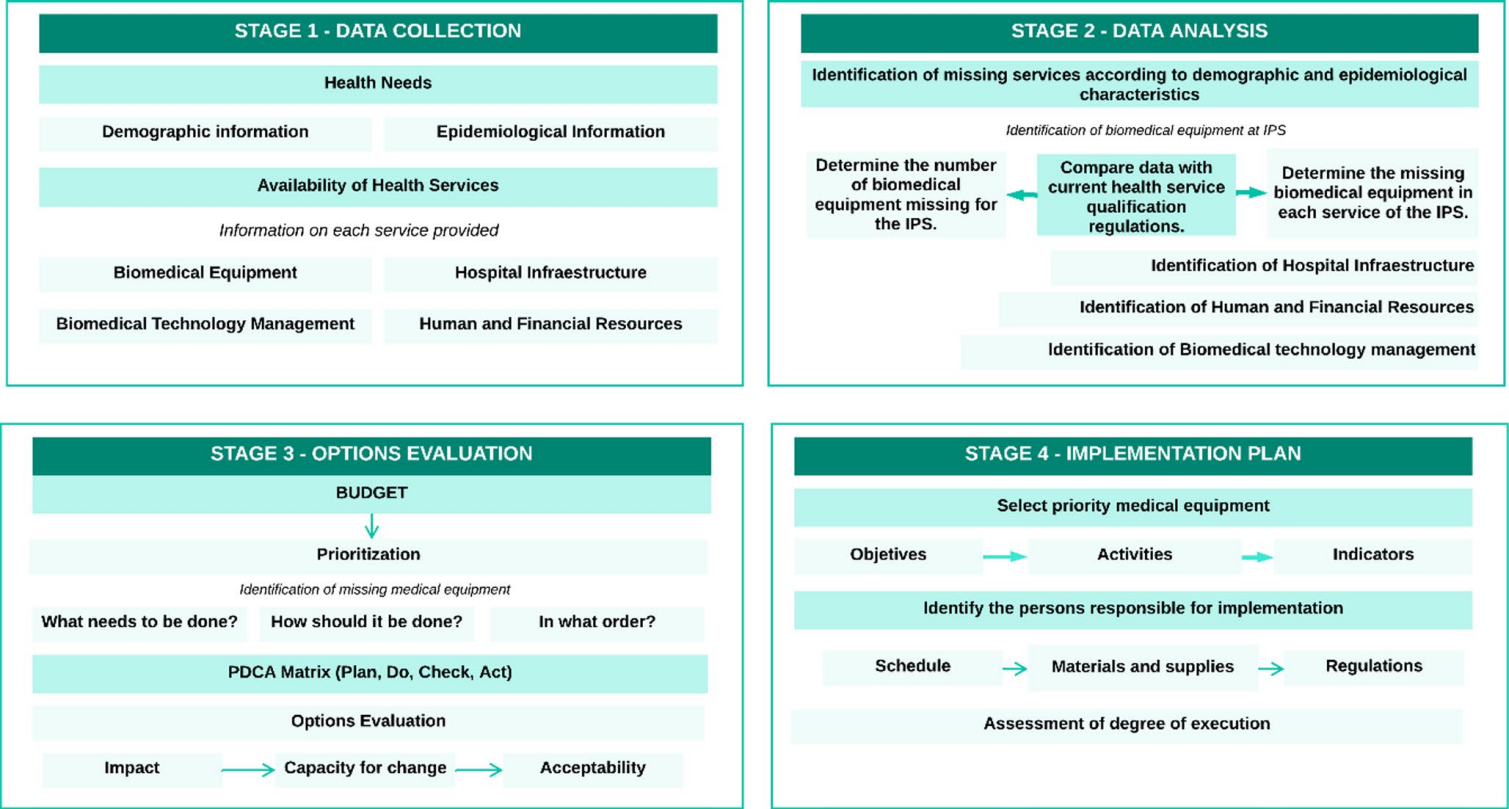
Needs assessment model for the acquisition of biomedical equipment –​​Source: Own elaboration

The proposed model was structured into four sequential and complementary stages: (1) data collection, (2) data analysis, (3) option evaluation, and (4) development of the implementation plan, as illustrated in Fig. 6. This structure responds to the need to align each methodological component with the criteria defined by the World Health Organization for medical device needs assessment, ensuring a systematic approach.

Each stage corresponds to specific tools developed and implemented in the web platform and validated in real settings. Stage 1, Data Collection, includes gathering demographic and epidemiological profiles and characterizing service supply, biomedical inventory, infrastructure, and human resources. Stage 2, Data Analysis, generates automated reports and matrices to identify service gaps, equipment needs, and renewal requirements. Stage 3, Option Evaluation, applies prioritization based on acquisition impact and resource availability, offering decision-making support. Finally, Stage 4, Implementation Plan Development, structures prioritized data into a format compatible with the Adjusted General Methodology (AGM), supporting the evaluation and scheduling of health investment projects.

This evolution toward a standardized model aligned with international benchmarks strengthens the applicability of the approach, promoting informed, efficient, and sustainable decision-making in biomedical equipment planning within healthcare institutions.

1. Model Validation in Healthcare Institutions

The model was validated in two IPS with different levels of complexity, enabling evaluation of its applicability across diverse operational contexts, including rural areas. In the medium-complexity IPS, a version developed in Excel with VBA was used, to standardize the diagnostic and biomedical equipment acquisition planning process. In the low-complexity IPS, a web-based version built with Laravel was implemented, assessing usability and efficiency in a real-world setting.

The validation process included both qualitative and quantitative usability assessments. The qualitative validation focused on expert perception, Users highlighted the ease of navigation, interface comprehension, and efficiency in data entry and analysis. Registration and system access were perceived as highly intuitive, and the time required for institutional characterization and demographic data entry ranged between 1 and 7 min, reflecting a low learning curve.

The system was rated highly efficient in report generation, facilitating structured planning for biomedical technology acquisition. A comparative analysis with national accreditation standards showed strong alignment with efficiency, usability, and technology management criteria. An external biomedical engineer validated the model, confirming its value for strategic decisions on equipment renewal and acquisition. These results, summarized in Table 7, demonstrate the model’s capacity to support institutions in assessing equipment needs, improving decisions, and aligning with health policies and technical standards.

**Table 7:** Key considerations of the validation process – Source: own elaboration

General Considerations		
Validation Methodology: The software model was validated in two healthcare settings.	**Medium-Complexity IPS**	Implemented using VBA and Excel to standardize the evaluation and acquisition of biomedical equipment.
	**Low-Complexity** **IPS**	A web-based platform developed in Laravel was tested in a rural healthcare setting to assess usability and efficiency.
Validation Process: Included both qualitative and quantitative usability assessments to measure user experience, ease of use, and task completion efficiency.	**Usability Assessment**	Conducted to evaluate user interaction with the system.
	**Qualitative** **Usability Testing**	Users provided feedback on ease of navigation, comprehension, challenges, and suggested improvements.
	**Quantitative Usability Testing**	Metrics collected included task completion rates, time required for data entry and analysis, and system efficiency in generating reports.
Results - Registration Process Users found registration straightforward, particularly when provided with predefined credentials.	**Login Experience**	Both users rated the login process as “Very Intuitive.”
	**Data Entry Efficiency**	IPS characterization took between 1 and 3 min, while demographic and epidemiological data entry required 2 to 7 min.
	**System Efficiency**	The model was rated as “Very Efficient” in generating reports on biomedical equipment needs.
Additional Considerations		
Implementation in a Real-World Setting	The validation was executed in a regional healthcare institution, where the model was applied to assess biomedical equipment needs.	
Healthcare Facility Characterization	Data collection was conducted to systematically identify biomedical equipment requirements and service gaps.	
Biomedical Equipment Acquisition Plan	A structured acquisition plan was developed based on the model’s analysis, prioritizing critical equipment needs.	
Comparative Analysis with Standards	The model was evaluated against national health accreditation criteria, demonstrating high alignment with efficiency and usability benchmarks.	
Expert Evaluation	A biomedical engineer assessed the model and confirmed its effectiveness in optimizing equipment acquisition and decision-making in healthcare settings.	
Overall Conclusion	The validation process confirmed that the software model effectively supports healthcare institutions in assessing and acquiring biomedical equipment, demonstrating usability, efficiency, and compliance with healthcare standards.	

Quantitative results were analyzed using descriptive statistics (mean ± standard deviation, 95% confidence intervals). To evaluate improvement before and after implementation, paired t-tests were applied to accreditation components GT1, GT2, and GT6, considering a significance level of α = 0.05.

The quantitative validation assessed the model’s compliance with accreditation standards using a 0-to-5 scale. It covered three components: GT1 (Planning, management, and evaluation of technology), GT2 (Acquisition, monitoring, and replacement policy), and GT6 (Technology renewal policy), with average scores of 4.40, 4.76, and 4.71, respectively. The model fully met the benchmarks for GT2 and GT6, whereas GT1 showed a 0.6-point gap, suggesting improvement potential. Evaluation time decreased by 70%, and expert review supported the model’s effectiveness in enhancing acquisition and service quality. These efficiency gains outweighed the moderate development and maintenance costs of the platform. Data are reported as mean ± standard deviation, with 95% confidence intervals calculated for each accreditation component. Comparative pre- and post-implementation analysis showed statistically significant improvement (*p* < 0.05) in GT2 and GT6.

**Fig. 7:**
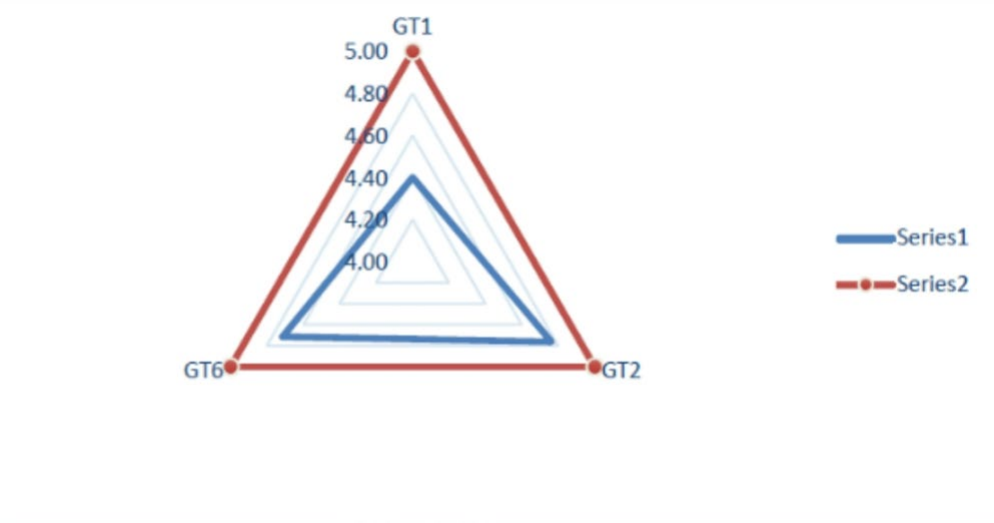
Quantitative Validation Results Compared to National Accreditation Standards

A visual summary of this assessment is shown in Fig. 7, prcomparing the model’s performance (Series 1) against the ideal score (Series 2) across the three accreditation dimensions.

## Discussion

The model automates analysis to detect equipment gaps in IPSs and supports compliance with accreditation standards. Such automation aligns with research promoting standardized evaluations to enhance healthcare quality [14].

Unlike traditional methodologies, this model includes demographic and epidemiological variables in equipment prioritization. It addresses shortages while aligning resources with service demands. This integration is key for effective healthcare planning and reducing inequities. Needs-based frameworks, such as those proposed by Birch et al. (2006), emphasize that incorporating such data enables a more comprehensive approach to resource allocation by estimating service flows and translating them into the necessary stock of providers [15]. The model also considers infrastructure, financial and human resources, and biomedical technology management, offering a context-specific approach essential to maintaining quality and efficiency, particularly in regions with disparities [16].

A key component of the model is its prioritization method based on the PDCA matrix (Plan, Do, Check, Act). This strategy supports the development of structured acquisition plans based on impact, feasibility, and acceptance, optimizing institutional investment. The PDCA cycle is a well-established framework for continuous improvement in hospitals, promoting a systematic approach to identifying needs, implementing actions, verifying outcomes, and standardizing effective processes [17]. The tool supports both MGA-based and directly funded acquisition projects.

Validation confirmed the model improves technology procurement processes. Broader institutional testing and user-driven refinements are encouraged. Multidisciplinary participation in health technology assessment (HTA) is vital for considering clinical, technical, and economic aspects, ensuring that selected technologies align with institutional goals [18, 19].

The model’s flexibility enables adaptation to institutions of varying complexity and geographic contexts. It is scalable and aligns with international standards. This adaptability is especially valuable where healthcare demands shift with population profiles. Integrating epidemiological data enhances responsiveness and supports informed decisions in resource allocation and technology assessment. This aligns with literature calling for the integration of epidemiology and health economics in health technology assessment processes [20].

Implementation may face challenges such as institutional resistance and limited staff training in technology management. By grounding the discussion in existing research, the model’s contributions are contextualized within the broader landscape of healthcare technology management, underscoring its relevance and potential impact. The implementation of the model in real-world settings revealed both technical and cultural barriers, including limited digital infrastructure and initial resistance to adopting standardized evaluation systems. Addressing these challenges through training and policy alignment is crucial for long-term sustainability. Although the model was validated in two institutions within a single Colombian region, the results demonstrated consistent performance and practical feasibility. These findings confirm the model’s practical value while highlighting the need for further validation.

In high-complexity hospitals, factors such as larger patient volumes, broader specialties, and more advanced infrastructure could influence its performance. Future studies should extend validation to additional institutions and healthcare systems to confirm scalability and robustness. Compared to traditional needs assessment methodologies, the proposed model combines international WHO standards, national regulatory alignment, and automated data analysis, providing a more structured and replicable framework for biomedical equipment planning [21].

Furthermore, differences in regulatory frameworks and interoperability of health data systems across countries may affect the model’s transferability. Future adaptations should integrate these contextual factors to ensure global applicability.

## Conclusions

The proposed model constitutes an innovative and structured approach for assessing and planning biomedical equipment needs in healthcare institutions (IPS). It consolidates technical, contextual, and regulatory factors into a cohesive digital framework. Its comprehensive design integrates demographic, epidemiological, infrastructure, human resource, and technological management variables, enabling transparent and context-based prioritization of acquisitions aligned with national quality and accreditation standards.

Quantitative validation confirmed consistency with national accreditation requirements, while qualitative evaluation—based on expert feedback—highlighted the model’s usefulness, efficiency, and usability as a decision-support tool. The integration of the PDCA cycle as a prioritization mechanism contributed to the formulation of sustainable technology acquisition plans aligned with institutional objectives, improving the planning process and resource allocation.

Nevertheless, the study was limited to two institutions in a single Colombian region, and it focused primarily on usability and planning performance rather than clinical, economic, or system-level outcomes. Broader validation—including high-complexity institutions and multidisciplinary perspectives—will be necessary to confirm the model’s scalability and generalizability.

While the model demonstrated adaptability to institutions of different sizes and contexts, its international applicability will depend on alignment with local regulatory frameworks and further real-world testing. Future integration with participatory health technology assessment processes may strengthen institutional adoption, sustainability, and equitable impact within healthcare systems.

## Data Availability

No datasets were generated or analysed during the current study.
